# Knowledge, Attitude, and Practice on Hygiene and Morbidity Status among Tertiary Students: The Case of Kotebe Metropolitan University, Addis Ababa, Ethiopia

**DOI:** 10.1155/2018/2094621

**Published:** 2018-08-29

**Authors:** Getachew Dagnew Gebreeyessus, Dessalew Berihun Adem

**Affiliations:** Department of Urban Environmental Management, Kotebe Metropolitan University, P.O. Box 31248, Addis Ababa, Ethiopia

## Abstract

Considerable fractions of the world's diseases are communicable, of which over 60% are infectious. Knowledge, attitude, and practice of hygiene are very important to decrease these disease burdens especially in places like higher education institutions. This study is aimed at revealing the status and gaps on knowledge, attitude, and practice of hygiene among Kotebe Metropolitan University students. Alongside, morbidity records of the students' clinic are reviewed. Sampled regular students who are boarding and who serve the students' canteen are studied. The investigation applied a cross-sectional study design. A structured questionnaire is administered following a pretest, and the data collected are analyzed using “SPSS v.20.” The review on morbidity record showed that the leading infections so far are respiratory (47%), gastrointestinal (amoebiasis, giardiasis, and typhoid) (34%), and eye and skin infections (16%). Regarding the responses to the knowledge questions, 1451 (60.8%) were correct while 934 (39.2%) were incorrect. Concerning handwashing as knowledge question, significant difference (*p* ≈ 0.00) existed between genders. Over 50% of the respondents do think parasitic infections typically amoeba is acquired not due to the contaminated vegetables, but rather they assume that such vegetables trigger those parasites already lodging in their gut. Above 60% of the respondents agreed that sharing drinking cups as a sign of affection as unfavorable attitude. In relation to that, the students' attitude highly varies by gender. However, the responses on hygiene practice enquiries appear to be promising. Further, the students do some practice while not having the desired level of knowledge on hygiene. Generally, there is a considerable gap in the knowledge, attitude, and practice of hygiene among students.

## 1. Introduction

The word hygiene is the practice of keeping oneself and one's surroundings clean, especially in order to prevent illness or the spread of diseases [1]. Hygiene practices are vital to one's health and well-being especially in the prevention of the communicable diseases. In this regard, it is obvious that in most developing countries including Ethiopia hygiene is important since hygiene preventable diseases are prevalent. These diseases account for 80% of the illnesses together with other infectious diseases and malnutrition [2]. In relation to that, students of higher education institutions (HEIs) in the country are targets of hygiene promotion [3]. For instance, a study by Aklilu et al. found that over 45% of the studied food handlers who work for the students' canteen in Addis Ababa University are positive for different intestinal parasites [4].

In that respect, water supply, hygiene, and sanitation are highly demanding to lower and avoid the burden of communicable diseases significantly [5]. Especially hand hygiene is considered as one of the most important infection control measures since it breaks the transmission of microorganisms especially in medical centers [6, 7]. An earlier study in India showed that faeco-oral transmission of enteric diseases is caused by inadequate handwashing after defecation. Moreover, the study identified that good handwashing behavior is associated with better socioeconomic indicators including education of women [8–10] especially when the right procedure for handwashing is strictly followed [11].

**Figure 1:**
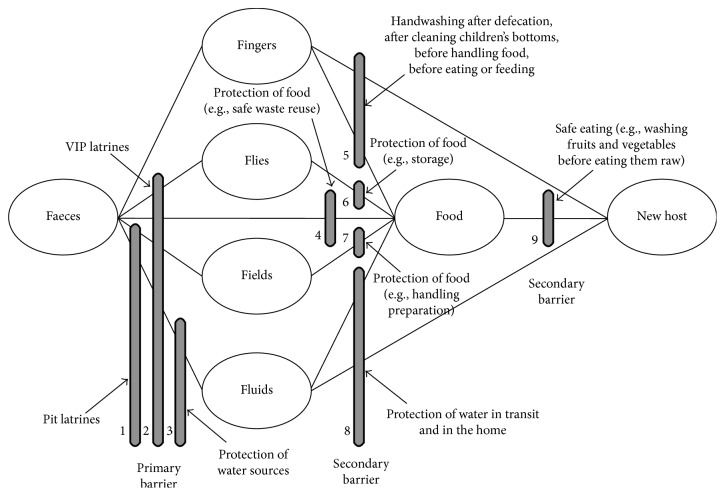
Diagram to depict the fecal-oral routes for disease transmission and the possible barriers [16].

The notion hygiene comes first with the importance of blocking fecal-oral route diseases transmission as portrayed in Figure 1, often known as the “F-diagram” [12]. According to studies, handwashing with soap and water can reduce diarrheal disease by 35% or more [13]. Handwashing can also help to reduce the prevalence of eye infections such as conjunctivitis and trachoma as well as respiratory illness significantly [14, 15]. According to Figure 1, it is possible to put about nine barriers to prevent or block fecal-oral route of disease transmission [16]. In addition, hygiene is very important to avoid contamination of water and soil by intestinal and other pathogens [12, 17, 18].

Further in history, religions connected hygiene with cleanliness and conduct. In this respect, understanding what hygiene meant in peoples' mind is crucial to bring desirable changes [19–21]. Therefore, all issues of hygiene demand the combination of proper knowledge, attitude, and practice (KAP) so that people can be knowledgeable about something, be convinced and practice it reasonably.

Proper hygiene cannot be ensured with the only provision of hygiene facilities [22]. Even though hygiene facilities are available, behavior of the users do matter in the effective control of those communicable diseases. Unless people are knowledgeable on the health risk posed for not practicing proper hygiene, it is likely that they ignore or under practice hygiene as evidenced by a related study conducted in Bangladeshi universities [23]. Therefore, KAP is a major challenge even though sanitation facilities could be well established as exhibited in countries like South Africa [24]. In that regard, hygiene related studies recommended the relevance of identifying gaps in KAP at HEIs [3].

In Ethiopian HEIs including Kotebe Metropolitan University (KMU), gaps in KAP result in a more serious effect since the students are living in a confined condition, sharing common services. Epidemiologically, common source health problems can affect a mass of people in short time thus the potential of the spread of infection is considerably high in such conditions with further impact on the students' academic development or achievement [25, 26].

Despite those huge impacts of hygiene, there is no history of hygiene-related study in KMU. Therefore, this study is aimed at investigating the KAP of KMU students, reviewing morbidity records in KMU, and identifying possible variables of hygiene communication for subsequent intervention.

## 2. Method and Materials

The study is conducted from May 2016 to October 2016 in order to include the summer and winter modality students at KMU. Initially, a retrospective data review was performed on morbidity record of the students at the campus clinic in KMU. This cross-sectional study is conducted using a structured questionnaire that is administered to these sampled students.

### 2.1. Study Area

**Figure 2:**
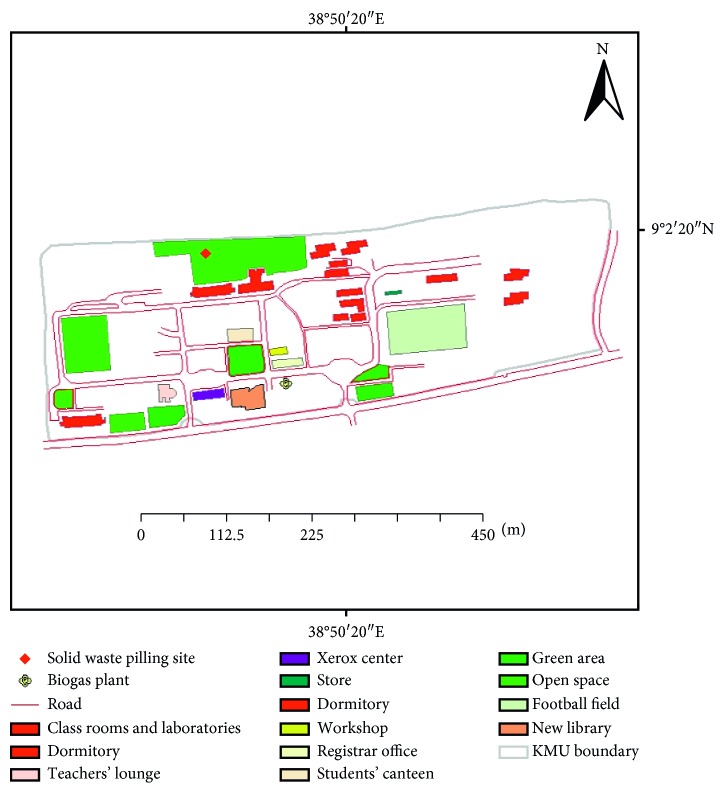
Scaled map of Kotebe Metropolitan University.

KMU is located within the territory of Addis Ababa city, and it is one of the expanding HEIs in Ethiopia. Currently, KMU is hosting over 10,064 students in diverse modalities, of whom 3634 are enrolled in the regular programs during the year 2015/2016. Regarding sanitation, in KMU, there are 68 sanitation and hygiene infrastructures according to the annual abstract of the university formerly known as Kotebe University College [27]. Spatially, the university is laid on little over 15 hectares of land (Figure 2) located to the east of the capital, Addis Ababa.

### 2.2. Sample Size

The sampling size is calculated using the Slovin's formula for sample size [28]:

(1) $$\begin{array}{c} n=\frac{N}{1+Ne^{2}}, \end{array}$$

where *n* = sample size, *N* = total population, and *e*^2^ = margin of error. Thus, *n*=3634/(1+3634  *∗*  (0.05)^2^)=360. In calculating the sample size, a 5% margin of error is considered.

### 2.3. Sampling

Regular students of KMU in the study period were eligible for the administration of the questionnaire. Proportional sampling with respect to the size of enrolled students per program is followed by random selection of each subject in a program just to ensure a representation of existing programs at KMU. Consequently, those boarding students serving the students' canteen were included. The structured questionnaire is administered first by asking questions of gender, age, and program of enrollment as sociodemographic variables. The number of morbidity reviewed was 343, and the number of interviewed subjects was 360, as specified in the above sampling.

### 2.4. Ethical Clearance

This study is conducted based on the acquisition of orally informed consent of the respondents and the ethical clearance obtained by the time from the Research and Community Service Directorate at KMU.

### 2.5. Data Processing, Analysis, and Interpretation

Excel is first utilized for encoding data sets. The data generated were then transferred to the “SPSS v.20” software for analysis. Excel was also used for graphing and tabulation. From Excel, the data and information were exported to word format to discuss and compare the finding with other related works. The chi-square test is applied to see the statistical significances (*p* < 0.05) among variables. The interpretability criteria with at least five items with significant loadings were considered to extract analyzable questions in each category of the KAP questions. Based on the explanatory analysis at 0.5 case loading, manageable number of questions from the KAP domain was selected for further statistical analysis.

A student who fails to correctly answer one or more of the selected questions in each category of investigation, KAP, is considered not knowledgeable, unfavorable attitude, and poor hygiene practicing. The report from this study will be disseminated to interested stakeholders through Internet and is submitted to the Research and Community Service Directorate of KMU.

### 2.6. Data Quality Control

The interview questions were checked for quality in a pretest and were cleaned. Before administering the questionnaire, consent and clarification of the purpose of the study were ensured. The questionnaires were administered face to face with data collectors. In fact, the data collectors were oriented about the procedures of administration.

## 3. Result

In this study, a sample size of 360 students were interviewed, and 100% of the subjects gave their responses. In addition to that, a yearlong morbidity records from KMU students' clinic were reviewed which are presented in the subsequent sections.

### 3.1. Situational Analysis on Hygiene and Morbidity

The morbidity data were anonymously reviewed for the period of October 2015 till August 2016. Gender and age data were reviewed (Tables 1 and 2).

**Figure 3:**
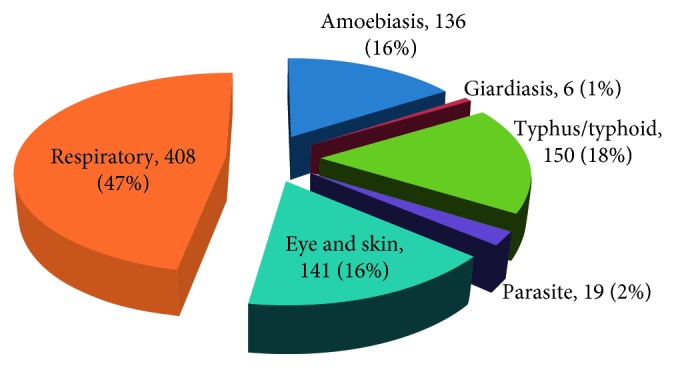
Morbidity record in KMU clinic.

Based on the morbidity review from the students' clinic, nearly half of the cases were respiratory followed by typhoid, amoeba, and eye and skin illnesses (Figure 3).

### 3.2. Demographic Condition of the Study Group

**Figure 4:**
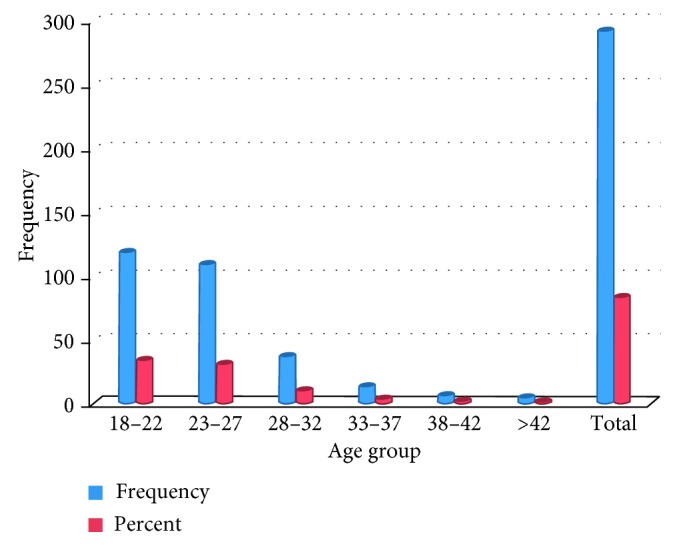
Age distribution of students (the numbers/percentages may not add up to the total number due to missing data).

The age distribution of the studied subjects shows that the minimum age is 19 and the maximum is 49 where the latter is due to the inclusion of adult in-service summer students (Figure 4).

The average age of the students was 24.7 years with a standard deviation of 5.5 years. Generally, some studies suggest that the age of an individual is expected to influence his/her hygiene practice or experience [29]. By gender, about 206 (59.5%) were male while the rest 140 (40.5%) were female students.

**Figure 5:**
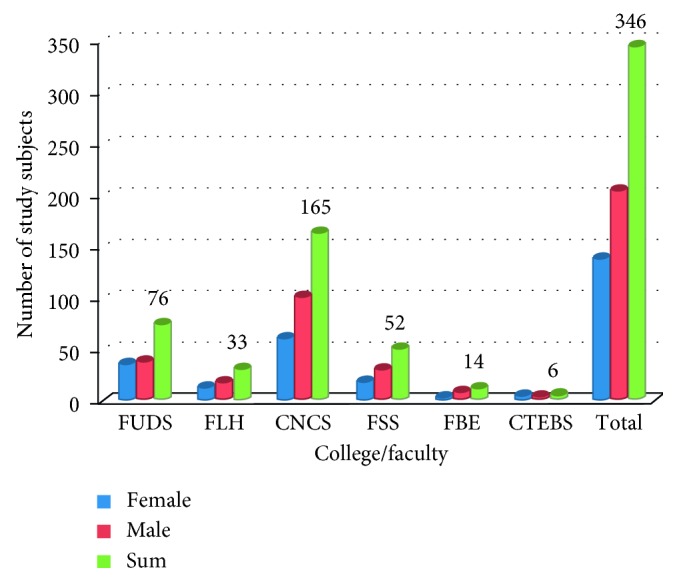
Distribution of the study subjects by faculty and sex (the numbers/percentages may not add up to the total number due to missing data).

Regarding the students' program distribution, due to the relatively largest number of students under the College of Natural and Computational Sciences (CNCS), nearly 50% of the study subjects belonged to CNCS by category, compared to the least study participants from the College of Education and Behavioral Studies (CEBS), as depicted in Figure 5.

### 3.3. The Knowledge Level of Students towards Hygiene

Seven out of 13 knowledge questions were analyzed based on priority and relevance regarding the study objectives and to enhance statistical interpretability [16]. Though it is impossible to judge the knowledge or attitude of respondents based on a single question from pedagogic perspective, there is no limit to the number of questions that should be presented. Thus this study applied the same approach as the published articles on KAP studies [5, 30]. Regarding the hygiene knowledge of the respondents, 150 (44%) were knowledgeable, that is, below average. According to Table 3, 1612 (68%) of the knowledge-related responses were correct while the rest 32% were not. In the table, three percentages may not add up to 100 in each question due to the missing data that some respondents may be reserved or escaped to respond few questions.

### 3.4. Attitude of Students towards Hygiene

According to the responses on those attitude questions, overall it is observed that 928 (56.2%) of the responses were favorable, while 724 (43.8%) of the responses were unfavorable (Table 4). However, it should be noted that only 115 (35%) fully responded the acceptable attitude based on high overall score by fully answering of the selected attitude questions, while the majority of them did not.

It would be interesting to appreciate the disparity between the sexes in each respective question regarding hygiene perception. Such kinds of epidemiological studies inevitably consider gender as variable simply because such studies will have social junction. Moreover, attitude-related studies are impacted by sex among other variables [25]. The attitude question “Sharing drinking cups is a sign of affection or liking one another” is not correctly answered by both sexes with an insignificant statistical difference (Table 5). In the other three of the attitude questions, however, a statistically significant difference is observed between the sexes (Table 6). Tables 5 and 6 present the chi-square test for those questions which are picked to illustrate statistically meaningful interpretation.

### 3.5. The Hygiene Practice of Students

From the 2283 responses (participants ∗ number of practice questions) to those practice questions, 1780 (78%) were answered correctly while the rest 503 (22%) are answered the wrong practice questions regarding hygiene (Table 7). Based on the selected and analyzed six practice questions, 240 (74%) of the respondents were aware of the desirable hygiene practices while the rest did not.

## 4. Discussion

Identifying gaps in KAP among HEI students is important not only to prevent or control epidemics [31] but also to control the nonepidemic and hygiene preventable cases. This study first reviewed the morbidity condition of regular program students in KMU before determining the KAP of students towards hygiene at KMU. Based on the morbidity survey, the three major groups of illness recorded are respiratory (47%), gastrointestinal (amoebiasis, giardiasis, and typhoid) (35%), and eye and skin infections (16%) sequentially (Figure 3). Figure 3 dictates the fact that the majority, if not all, are communicable and hence are potentially avoidable with some level of hygiene KAP along with safe supply of services on a community level [4]. Nearly half (408 cases) of the morbidity in that same period were due to respiratory problems alarming the conditions of overcrowding in dormitories which demands attentions. Moreover, due to degree of contagiousness, typhoid [32] and typhus need significant intervention so that the university community should put hygiene as a priority area of awareness and do practice raising campaigns so as to break the routes of disease communication.

The result of this KAP study showed that there existed a significant gap among KMU students which needs attention for prompt intervention. Despite who the individual is in each respective knowledge question, it is possible to say that only 44% of the respondents do lack the basic knowledge on hygiene. Since failure to know what is necessary to maintain health by few is a threat to all, this percentage is of a significant value to trigger an awareness-raising intervention [22]. Particularly, the knowledge or even implied in attitude towards amoeba infection is sensitive. Over half of the study subjects do think that amoeba is not caused by eating contaminated raw vegetable (Table 2). Similarly, the participants' knowledge on the mechanical involvement of cell phone on disease transmission is poor. This latter fact has implication on daily touching of cell phones while eating as it is often observed. The only exception from the knowledge question is a statistically significant dissimilarity between male and female respondents to the question on handwashing (*p* ≈ 0.00). Nearly half of the female respondents think that handwashing after meal is more important compared to only one-fourth of the male students.

However, the knowledge of respondents at KMU towards hand hygiene looks good compared to other studies [7] as it is slightly over the developing countries' average which is reported to be only 14% [33]. Since hand hygiene is a very important measure to break disease transmission path against many respiratory and gastrointestinal infections [7], the gap between the male and female students in knowledge should still be narrowed. At the same time, it should be considered that washing hands using the right procedure is an issue to be effective against the control of communicable infectious diseases [34].

The response to attitude questions in this study signals the need for enabling conditions to control communicable diseases (Table 5). According to Table 7, a significantly diverging number of female students imperfectly perceive the health risk of contaminated water and the health benefit of bathing as well as the community health implication of open defecation. Thus, it is important to identify the actual KAP gaps to achieve the desirable behavioral change, especially changing the hygiene attitude of HEI students—as tomorrow's leaders—is crucial. Conversely, both sexes showed the necessary attitude towards perceiving the health risk that can be possible from mothers' hand hygiene deficiency on a statistically insignificant disparity (*p* value 0.254).

In spite of the knowledge and attitude questions and responses, the responses on hygiene practices appear promising. For instance, the hand hygiene practice is better than the hand hygiene knowledge of the students. This suggests that people could practice hygiene intuitively to a certain level. Alternatively, it can also be assumed that people could practice hygiene simply because they are used to or might have just inherited the practice from their parents or colleagues [35, 36]. In addition, it should be noted that washing hand with or without soap and doing it the right way matters. On the contrary, it should be remembered here that the availability of sanitary material and water as well as sanitation facility can limit significantly hygiene practice that if a disparity between knowledge or attitude and practice can be expected [23].

Though the wrong practical response (26%) is considerable to trigger an intervention in this study context, those who are practicing hygiene in their daily life should get to know why and how they are doing it. Despite the relatively lower score in attitude and knowledge regarding hygiene, the practice is better compared to other studies [37]. However, the implication of failure to perform a single hygiene practice cannot be underestimated. For instance, people can lower or avoid viral infection like glandular fever, fungal infection like ring worm, and bacterial infection including the scarlet fever by avoiding sharing of items including drinking utensils, towels, spoons, and forks [38, 39] that all require progress in an acceptable attitude.

Furthermore, all the responses, except the question on whether they brush their teeth regularly or not, on the selected hygiene practice questions did not show a statistically significant difference between the males and the females on a 95% significance level. Both sexes on average showed difference in teeth washing practice in favor of female students on a 95% confidence level (*p* ≈ 0.03). However, oral hygiene performance of KMU students is slightly lower compared to related studies by about 8% or more [40, 41].

## 5. Limitations

Although this study attempted to determine the “soft” component of sanitation and hygiene by administering the questionnaire on KAP and by reviewing the morbidity record at the students' clinic in a certain period, the students are not the only players of environmental and public health in HEIs. Therefore, other role players including the determination of the status of hygiene and sanitation services and awareness of officials are equally important which is unaddressed in this paper.

## 6. Conclusion

Based on the morbidity data reviewed, the leading diseases prevailing in KMU are preventable by some level of hygiene practices. Based on this study, there exists a considerable gap in KAP on hygiene among KMU students. These gaps are significant to trigger intervention either on awareness raising sessions or other supervision activities. Regarding attitude-related responses, the students showed significant disparity between gender, and somehow there also existed difference in practice and knowledge. Therefore, the progress towards hygiene solutions has to consider this gender disparity in order to be effective. Further detailed studies including the sanitary service adequacy and hygiene information communication is recommended.

## Figures and Tables

**Table 1:** Sex distribution of students diagnosed in KMU clinic.

Gender	Frequency	Percent
Female	128	37.3
Male	215	62.7
Total	343	100

**Table 2:** Age and sex distribution of students diagnosed in KMU clinic.

Age group	Frequency	Percent
17–22	249	72.6
23–27	61	17.8
≥28	33	9.6
All	343	100

**Table 3:** Frequency of the response to the selected knowledge questions.

No.	Knowledge questions and answers		Frequency	Percent	No valid cases
1	Sharing drinking cups without washing brings health problem	Yes	285	82.4	345
		No	60	17.3	
2	Handwashing with or without soap is the same	Yes	73	21.1	343
		No	270	78.0	
3	Eating raw vegetables does not cause amoebic infection, it rather aggravates the latent form already existing in our gut	Yes	174	50.3	342
		No	168	48.6	
4	Sitting in a room with windows open can avoid transmission of respiratory infection	No	94	27.2	341
		Yes	247	71.4	
5	Washing hands after meal is more important than doing it before meal	Yes	114	32.9	
		No	225	65.0	339
6	Human feces contain germs that can cause infection	Yes	263	76.0	336
		No	73	21.1	
7	Electronic media like cell phones can mechanically communicate diseases	Yes	154	44.5	337
		No	183	52.9	

**Table 4:** Response frequencies to the selected attitude questions.

No.	Attitude questions and answers		Frequency	Percent	Number of valid cases
1	Bathing is more important for beauty purpose than for health	Yes	148	42.8	340
		No	192	55.5	
2	Open defecation is more of privacy issue than it is hygiene and environmental	Yes	194	56.1	320
		No	126	36.4	
3	Sharing drinking cups is a sign of affection or liking one another	Yes	214	61.8	330
		No	116	33.5	
4	Mothers' hand does not cause problem even though it is unhygienic	Yes	69	19.9	331
		No	262	75.7	
5	I agree with the saying that “Nothing is bad with a mother and water”	Yes	99	28.6	331
		No	232	67.1	

**Table 5:** Cross tab on sex and an attitude question on sharing cups.

Sharing drinking cups is a sign of affection or liking one another			Chi-square tests			
Count	Yes	No		Value	df	Asymp. sig. (2-sided)
Female	88	49	Pearson chi-square	3.322	2	0.190
Male	126	67	Likelihood ratio	3.659	2	0.160

**Table 6:** The chi-square test on the selected attitude questions with respect to gender.

No.	Attitude question	Sex	Frequency			*p* value
			Yes	No	Total	
1	I agree with the saying that “Nothing is bad with a mother and water”	Female	50	88	140	0.009
		male	49	144	206	
2	Bathing is more important for beauty purpose than for health	Female	75	62	140	0.003
		male	73	130	206	
3	Open defecation is more of privacy issue than it is hygiene and environmental	Female	90	42	140	0.039
		male	104	84	206	

**Table 7:** Frequency of responses to the selected questions on hygiene practice.

No.	Practice questions		Frequency	Percent	Number of valid cases
1	Do you usually wash your hands before meal?	Yes	268	77.5	327
		No	59	17.1	
2	Do you usually wash your hands after meal?	Yes	253	73.1	340
		No	77	22.3	
3	Do you regularly clean your teeth?	Yes	248	71.7	326
		No	78	22.5	
4	Do you always wash your hands after defecation?	Yes	269	77.7	318
		No	49	14.2	
5	Do you often trim your nails?	Yes	254	73.4	326
		No	72	20.8	
		No	206	59.5	
6	Do you wash your feet every day?	Yes	282	81.5	329
		No	47	13.6	

## Data Availability

The data used to support the findings of this study are available from the corresponding author upon request.
